# 20-year experience with pediatric heart transplant in a developing country

**DOI:** 10.1186/1749-8090-8-S1-O309

**Published:** 2013-09-11

**Authors:** G Liguori, J Penha, L Miana, L Caneo, C Tanamati, M Jatene

**Affiliations:** 1 Heart Institute (InCor), University of Sao Paulo Medical School, Sao Paulo, Brazil

## Background

Cardiomyopathies in advanced stages and highly complex congenital heart disease may not be amenable to therapy or surgical correction thus being indicated pediatric heart transplant. In this study, we sought to report and evaluate the immediate and long-term outcomes of our 20 years experience in pediatric heart transplant.

## Methods

We conducted a retrospective study of 106 patients undergoing pediatric heart transplant between 1992 and 2011. We analyzed medical records, surgical reports and results of complementary tests.

## Results

The mean age at transplantation was 5.9 years (min=12 days, max=18 years). The preoperative diagnosis was cardiomyopathy in 79% and congenital heart disease in 21%. The surgical technique for all cases was orthotopic transplantation, having been used the biatrial method until 1996 and, since then, the bicaval method. There were four retransplantation, three due to hyperacute rejection and one due to graft vasculopathy. Survival was 84% during hospital stay, 81% in 1 year, 72% in 5 years, 62% in 10 years and 56% in 15 years. Among the postransplant comorbidities, recurrent lung infection was present in 70%, hypertension in 30%, graft vasculopathy in 25% and lymphoproliferative disease in 10%. During follow-up, one patient underwent renal transplantation 16 years after heart transplantation.

## Conclusion

Our 20 years experience in pediatric heart transplant present results compatible to the literature, with values of survival and morbidity that strengthen it as an alternative to treat different heart diseases of childhood.

